# Modulations in motor unit discharge are related to changes in fascicle length during isometric contractions

**DOI:** 10.1152/japplphysiol.00758.2021

**Published:** 2022-10-13

**Authors:** Eduardo Martinez-Valdes, Francesco Negro, Alberto Botter, Patricio A. Pincheira, Giacinto Luigi Cerone, Deborah Falla, Glen A. Lichtwark, Andrew G. Cresswell

**Affiliations:** ^1^Centre of Precision Rehabilitation for Spinal Pain, School of Sport, Exercise and Rehabilitation Sciences, University of Birmingham, Birmingham, United Kingdom; ^2^Department of Clinical and Experimental Sciences, Università degli Studi di Brescia, Brescia, Italy; ^3^Laboratory for Engineering of the Neuromuscular System (LISiN), Department of Electronics and Telecommunication, Politecnico di Torino, Torino, Italy; ^4^PoliToBIOMed Lab, Politecnico di Torino, Turin, Italy; ^5^School of Human Movement and Nutrition Sciences, The University of Queensland, Brisbane, Queensland, Australia

**Keywords:** EMG, motor unit, muscle contraction, neuromechanics, ultrasound

## Abstract

The integration of electromyography (EMG) and ultrasound imaging has provided important information about the mechanisms of muscle activation and contraction. Unfortunately, conventional bipolar EMG does not allow an accurate assessment of the interplay between the neural drive received by muscles, changes in fascicle length and torque. We aimed to assess the relationship between modulations in tibialis anterior muscle (TA) motor unit (MU) discharge, fascicle length, and dorsiflexion torque using ultrasound-transparent high-density EMG electrodes. EMG and ultrasound images were recorded simultaneously from TA using a 32-electrode silicon matrix while performing isometric dorsiflexion contractions at two ankle joint positions (0° or 30° plantar flexion) and torques (20% or 40% of maximum). EMG signals were decomposed into MUs and changes in fascicle length were assessed with a fascicle-tracking algorithm. MU firings were converted into a cumulative spike train (CST) that was cross-correlated with torque (CST-torque) and fascicle length (CST-length). High cross-correlations were found for CST-length (0.60, range: 0.31–0.85) and CST-torque (0.71, range: 0.31–0.88). Cross-correlation delays revealed that the delay between CST-fascicle length (∼75 ms) was smaller than CST-torque (∼150 ms, *P* < 0.001). These delays affected MU recruitment and de-recruitment thresholds since the fascicle length at which MUs were recruited and de-recruited was similar but MU recruitment-de-recruitment torque varied. This study demonstrates that changes in TA fascicle length are related to modulations in MU firing and dorsiflexion torque. These relationships allow assessment of the interplay between neural drive, muscle contraction and torque, enabling the time required to convert neural activity into movement to be quantified.

**NEW & NOTEWORTHY** By employing ultrasound-transparent high-density EMG electrodes, we show that modulations in tibialis anterior muscle motor unit discharge rate were related to both changes in fascicle length and resultant torque. These relationships permitted the quantification of the relative delays between fluctuations in neural drive, muscle contraction, and transfer of torque via the tendon during sustained isometric dorsiflexion contractions, providing information on the conversion of neural activity into muscle force during a contraction.

## Graphical abstract

**Figure:**
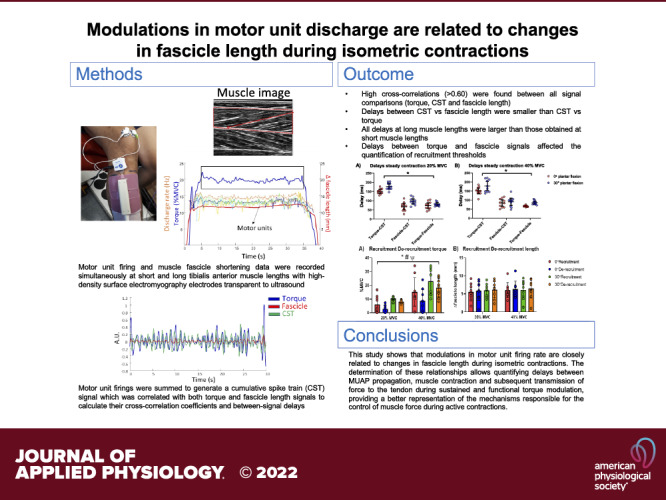

## INTRODUCTION

One of the most fundamental issues in motor control is to understand how the nervous system interacts with muscles for the generation of appropriate joint torques and control of movement. Although some answers have been obtained from studies in the fields of neurophysiology and biomechanics, limitations in previous recording techniques mean they have not generated clear information on how neural activity is influenced by muscle mechanics and vice-versa (1, 2). The recent integration of electromyography (EMG) recordings and ultrasound imaging has provided important information about the relationships between muscle activation and contraction (3–7). These techniques have helped to determine the level of muscle activity related to a given change in fascicle length and establish the relationships between active/passive tissue mechanics (i.e., muscle and tendon compliance) and muscle activity. However, there are limitations of the current approaches to link muscle mechanics with neural drive [number and rate of motor neuron potentials received by muscles (8, 9)]. For example, numerous studies have used amplitude estimates from bipolar surface EMG recordings to assess changes in neural activity and the resultant force produced by muscles (10–13). Due to factors such as cross talk, amplitude cancellation, and underlying changes in muscle length and velocity, surface EMG amplitude is often poorly correlated with the resultant force produced by muscles and therefore cannot be used to directly understand the neural determinants of muscle contractions (14–16). Furthermore, studies that have directly examined changes in muscle architecture using ultrasound (5, 6, 17, 18) rarely image the same region of muscle from where the EMG is recorded (19). Therefore, changes in muscle architecture or morphology, or both, from nearby or more distal regions could potentially provide results that are not related to those from the region of interest. A few studies have attempted to address this issue by using fine-wire electromyography in close proximity to the ultrasound probe to assess motor unit behavior in relation to contraction dynamics (20–22). However, due to the high selectivity of fine-wire EMG, this technique only samples a relatively small muscle region, resulting in relatively low yields of motor units (23), thereby limiting the ability to accurately correlate the neural drive received by muscles to the mechanical output.

Ultrasound translucent high-density EMG (HDEMG-US) electrodes have been developed to enable the simultaneous recording of large numbers of motor units and ultrasound images from the same region of muscle during contractions (24). This technique has the potential to improve our understanding of the neuromechanical determinants of muscle force. However, this method has yet to be used to relate motor unit discharge characteristics with fascicle dynamics tracked from ultrasound images.

Our primary aim was to assess how tibialis anterior muscle (TA) motor unit firing characteristics modulate in relation to changes in TA fascicle length at different joint positions (short vs. long muscle lengths) and at different target dorsiflexion torques (20% and 40% of a maximum voluntary contraction, MVC). We examined the relationship between modulations in the motor unit cumulative spike train (CST) and changes in fascicle length and dorsiflexion torque. We hypothesized that modulations in the CST would be strongly correlated with changes in fascicle length, which would allow delays between motor unit firing, muscle contraction, and the torque transmitted via the tendon to be quantified. In addition, we also examined differences in recruitment threshold in relation to fascicle length and torque, as possible delays between fascicle shortening and the torque produced at the level of the tendon might affect the interpretation of motor unit recruitment and de-recruitment thresholds.

## METHODS

The study and all procedures were approved by the University of Queensland ethical committee (Approval Number: 2019001675) and were conducted in accordance with the Declaration of Helsinki. Ten healthy young male volunteers participated [age: mean (SD) 29 (5) yr]. Participants were excluded if they had any neuromuscular disorder, musculoskeletal injury (i.e., muscle strain), any current or previous history of lower limb pain/injury and aged <18 or >35 yr. Participants were asked to avoid any strenuous activity 24 h before testing. All participants gave written, informed consent.

### Experimental Setup and Task

**Figure 1.**
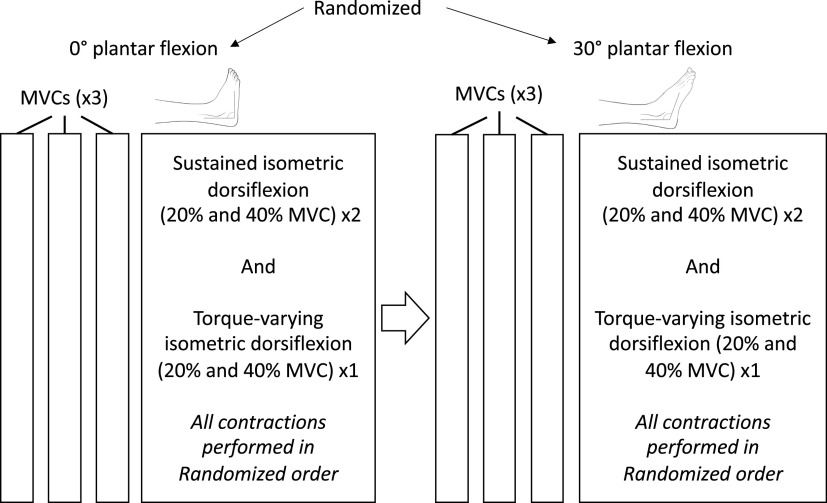
Study schematic. The order of the muscle-tendon lengths (0° and 30° of plantarflexion) and contractions performed at each length (sustained and torque-varying dorsiflexion at 20% and 40% MVC) was randomized. However, the randomization order was kept constant across muscle-tendon lengths. MVC, maximum voluntary contraction torque.

Participants were seated in a reclined position on the chair of an isokinetic dynamometer (Humac Norm, CSMi Computer Sports Medicine, Stoughton, WI). The right leg (dominant for all participants) was extended and positioned over a support with the knee flexed to 10° (with 0° representing full knee extension). To quantify ankle dorsiflexion torque the foot was securely strapped to the dynamometer with the approximate center of rotation of the ankle joint (lateral malleolus) aligned to the axle of the dynamometer. Participants performed sustained and torque-varying (sinusoidal) isometric dorsiflexion contractions at different torque levels at short (ankle at 0° plantar flexion, defined as the sole of the foot being perpendicular to the shank) and long TA lengths (ankle at 30° plantar flexion). A schematic describing the setup and measurements is shown in Fig. 1. The session began with participants’ performing three isometric ankle dorsiflexion MVCs at each ankle angle, where each MVC was separated by 2 min of rest. The order in which the ankle was positioned (short- and long TA lengths) was randomized. Then, after 5 min of rest, participants performed ramp-hold (sustained) and torque-varying sinusoidal contractions at 20% or 40% MVC. First, participants practiced performing brief ramp-hold contractions at low torque levels (20% MVC) with visual feedback of their exerted torque displayed on a computer monitor. For sustained isometric contractions, participants were asked to increase torque production at a rate of 10% MVC/s, then hold the contraction at the target torque for 30 s, and decrease torque at a rate of 10% MVC/s. Two sustained 20% MVC and two sustained 40% MVC contractions were performed. For the torque-varying sinusoidal isometric contractions, participants were again asked to increase and decrease torque production at a rate of 10% MVC/s at the beginning and end of the contraction. However, when target torque was reached, participants had to track a sinusoidal torque target at a frequency of 0.5 Hz and amplitude of 5% MVC. That is, torque was required to vary from 17.5% MVC to 22.5% MVC for the 20% MVC trials and 37.5% MVC to 42.5% MVC for the 40% MVC trials. One sinusoidal contraction was performed per torque level. Two minutes of rest were given between each contraction (sustained and sinusoidal). Sinusoidal contractions were used to test the ability of both the motor unit decomposition and fascicle tracking algorithms to identify motor units and track changes in fascicle length in conditions where greater variability in motor unit firing and fascicle length would occur. All contractions at each ankle angle were randomized, however, the randomization order of these contractions was kept constant between 0° and 30° of plantar flexion [i.e., if at the long muscle length (30° of plantar flexion), a 20% MVC sinusoidal contraction was performed first, then this was also the first contraction type performed at the short muscle length (0° of plantar flexion), see Fig. 1].

### Electromyography

**Figure 2.**
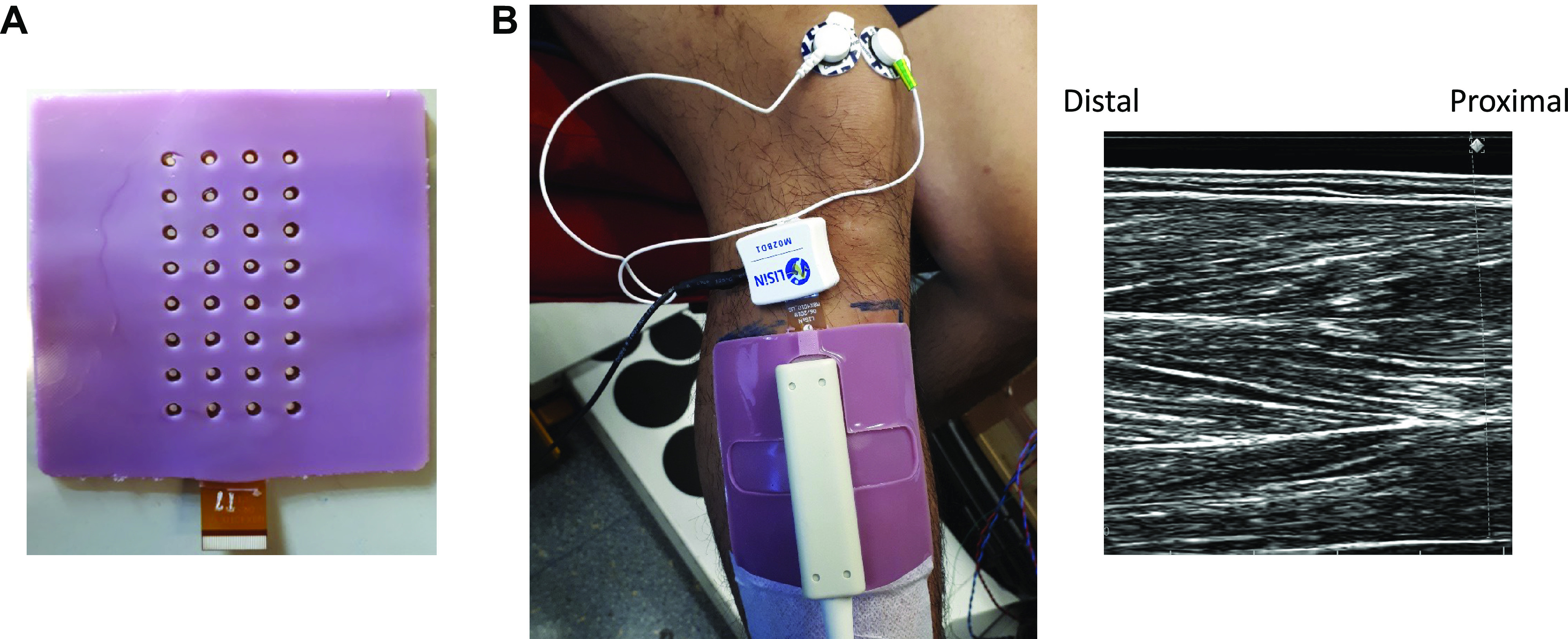
High-density surface electromyography (HDEMG) ultrasound-transparent electrodes. *A*: 8 × 4, 32-channel (10-mm interelectrode distance) electrode grid. *B*: HDEMG electrode grid with 32-channel HDEMG amplifier (connected on top of the electrode) and flat ultrasound probe can be seen on the left. Ultrasound image of proximal tibialis anterior muscle can be seen on the right. Note the quality of the image with accurate visualization of fascicles and, superficial, intermediate, and deep aponeuroses (*n* = 1, male participant).

Surface EMG signals were recorded from the TA using a high-density, 32-channel, HDEMG-US electrode grid (LiSIN, Torino, Italy) (24). Each grid consists of 8 × 4 electrodes (1-mm diameter, 10-mm interelectrode distance in both directions) embedded into a layer of silicon rubber (Fig. 2*A*). The array was located centrally between the proximal and distal tendons of the muscle with the columns aligned in the direction of the fascicles as confirmed by ultrasound imaging. Skin-electrode contact was made by inserting conductive gel (Sonogel, Bad Camberg, Germany) into the electrode cavities with a pipette (Eppendorf, Hamburg, Germany) (24). Signals were amplified and recorded using a wireless wearable HDEMG amplifier that contained a 16-bit analogue-digital converter (25) (Fig. 2*B*), connected directly to the electrode grids. Activity was recorded in monopolar mode at a sampling frequency of 2,048 Hz, with a gain of 192 ± 1 V/V and a bandpass filter with cut-off frequencies of 10 Hz and 500 Hz. Torque was also sampled at 2,048 Hz and recorded through the auxiliary input of the HDEMG amplifier. All EMG and torque data were processed and analyzed offline using MATLAB 2019b (MathWorks Inc., Natick, MA).

### Ultrasonography

Ultrasound images of TA fascicles were captured using B-mode ultrasonography (6 MHz, 80 frames/s, 60 mm field of view) using a 128-element multi-frequency transducer (LF9-5N60-A3; Telemed, Vilnius, Lithuania) attached to a PC-based ultrasound system (ArtUs EXT-1H scanner; Telemed, Vilnius, Lithuania). A dry HDEMG-US grid (without conductive gel) was first used to find the best alignment of the probe for clear visualization of TA fascicles when the probe is placed over the electrodes. Once the optimal position was identified the skin was marked with an indelible pen. The HDEMG-US electrode grid, with conductive gel added, was then positioned over the muscle at the same location and with the same orientation. The flat-shaped ultrasound transducer was then placed over the electrode grid and firmly strapped over the leg using an elastic bandage to prevent any movement. An example of the setup and an ultrasound image can be seen in Fig. 2*B*.

### HDEMG and Ultrasound Synchronization

Torque, HDEMG, and ultrasound data were synchronized utilizing an analogue pulse sent from an analog-digital board (Micro 1401-3, Cambridge Electronic Design, Cambridge, UK). The trigger signal was set at 80 Hz to control the frame-by-frame recording of the ultrasound. The same trigger signal was sent to the auxiliary input of the HDEMG device and was aligned offline with the tracked fascicle data (see *Concurrent Motor Unit and Fascicle Length Analysis* section) obtained from the ultrasound files.

### Motor Unit Decomposition and Fascicle Length Tracking

The HDEMG signals were decomposed into motor unit spike trains using a validated blind-source separation algorithm that automatically identifies the activity of multiple single motor units (26). Each identified motor unit was assessed for decomposition accuracy with a validated metric (Silhouette, SIL) that represents the accuracy of the decomposed spike train. Briefly, SIL is a normalized measure of the relative height of the peaks of the decomposed spike trains with respect to the baseline noise. Previous studies have shown that SIL is correlated to the rate of agreement with the two-source validation method (16). The identification of motor unit activity with HDEMG-US 32-channel (10 mm interelectrode distance) grids is more challenging compared with conventional 64-channel electrode grids (8 mm interelectrode distance), due to their lower selectivity and lower spatial sampling. Therefore, an accuracy level of 0.86 SIL (86% of accuracy) was used to approve or discard motor units, as previous studies have suggested that a less conservative threshold (>0.8) is required in more complex recordings (27, 28) [usual threshold is set at 0.90 SIL (26, 29)]. Missing pulses producing nonphysiological firing rates i.e., inter-spike intervals >250 ms, were manually and iteratively excluded and the pulse train was re-estimated to correct its firing frequency. In cases where the algorithm incorrectly assigned two or three pulses to what was likely only a single discharge time, the operator removed this firing and the final pulse trains were re-estimated as presented previously (30, 31). After this procedure, the average SIL value increased to 0.90 [standard deviation (SD): 0.007].

Changes in TA fascicle length were tracked offline with custom-made software (32) based on the validated Lucas–Kanade optical flow algorithm with affine transformation (33, 34). From each trial, we selected the fascicle in which we were able to visualize ∼80% of its length within the field of view of the image. We selected a fascicle from the superficial compartment of TA because *1*) this was the region of motor unit activity that was likely covered by the HDEMG electrode (35) and *2*) our preliminary results showed that changes in fascicle length from this region show a cross-correlation coefficient of at least 0.70 with the resultant torque output (see results). Changes in fascicle length were analyzed according to the amount of fascicle shortening exhibited during the contraction [Δ fascicle length (i.e., difference in fascicle length from rest to target torque (20% MVC))], to *1*) understand the amount of fascicle shortening required to recruit a motor unit and reach the required torque level (20% MVC and 40% MVC), and *2*) to be able to correlate fluctuations in fascicle length with fluctuations in motor unit discharge rate, as absolute changes in fascicle length are in an opposite direction to that of discharge rate, that is, fascicle length decreases when discharge rate increases. We also estimated pennation angle as the angle between the selected fascicle and its insertion into the central aponeurosis (36). The pennation angle was calculated when the muscle was at rest and when the torque was stable during the isometric contractions.

### Concurrent Motor Unit and Fascicle Length Analysis

For sustained isometric contractions, mean discharge rate was calculated from the time when torque became stable at the required level (plateau region). Motor unit recruitment and de-recruitment thresholds were defined as the ankle dorsiflexion torques (%MVC) or fascicle shortening lengths (Δ fascicle length, mm) at the times when the motor units commenced and stopped discharging action potentials. Recruitment threshold was determined during the ramp-up phase of the contraction and de-recruitment threshold was determined during the ramp-down phase of the contraction. We also tested the possibility of tracking the same motor units across the two different target torques and muscle lengths using a method based on cross-correlation of two-dimensional (2-D) motor unit action potentials (MUAPs) (37). In this procedure, matched MUAPs between the two trials (i.e., 20% MVC-0° vs. 20% MVC-30°) were visually inspected and the two identified motor units were regarded as the same when they had a cross-correlation coefficient >0.80 (37). Cross-correlation analysis was also used to assess similarities and delays between fluctuations in torque, fascicle length, and motor unit firing activity. Motor unit discharge times were summed to generate a cumulative spike train (CST) as done previously (38). In simple terms, the CST represents the cumulative activity of multiple motor units. The signals obtained from changes in fascicle length were interpolated to 2,048 Hz to match both CST and torque data. After these procedures, the signals obtained from CST, fascicle length, and torque were lowpass filtered (4th order zero-phase Butterworth, 2 Hz) and then highpass filtered (4th order zero-phase Butterworth, 0.75 Hz) as presented previously (39–41). These filtered signals were then cross-correlated to assess similarities in their fluctuations (cross-correlation coefficient) and to calculate the delays (calculated from the lags obtained from the cross-correlation function) between CST versus torque, CST versus fascicle length, and fascicle length versus torque. The cross-correlation coefficient between signals was calculated in 5-s segments with 50% overlap. The average cross-correlation coefficient and delay obtained from these segments (typically 11 to 12 segments) is reported.

### Statistical Analysis

Unless otherwise indicated, results are expressed as mean and SD. Normality of the data was assessed with the Shapiro–Wilk test and Sphericity was tested with the Mauchly test. Differences between fascicle parameters (absolute changes in length and pennation angle) at short- and long muscle lengths during rest were assessed with paired *t* tests. Absolute differences in fascicle length and pennation angle during isometric contractions were assessed with a two-way repeated measures analysis of variance (ANOVA), with factors of muscle length (0° or 30° of plantar flexion) and torque (20 or 40% MVC). All motor unit data obtained from each individual was averaged and then compared across individuals. For motor unit and Δ fascicle length data during isometric contractions, the following statistical tests were used: *1*) two-way repeated measures ANOVA with factors of muscle length and torque to assess differences in motor unit discharge rate, *2*) three-way repeated measures ANOVA with factors of muscle length, torque, and comparison of the generated signals (signal comparison: CST vs. torque, fascicle length vs. CST, and torque vs. fascicle length) to assess differences in cross-correlation results (correlation coefficient and delay), *3*) three-way repeated measures ANOVA with factors of muscle length, torque, and mean recruitment threshold (recruitment vs. de-recruitment) to assess differences between recruitment and de-recruitment thresholds expressed in terms of %MVC torque and Δ fascicle length. Finally, all cross-correlation results obtained during sustained and sinusoidal contractions were averaged for each contraction type and compared by paired *t* tests. All ANOVA analyses were performed using STATISTICA 12 (Statsoft, Tulsa, OK) and followed by pairwise comparisons with Bonferroni corrected *t* tests when significant. Statistical significance was set at *P* < 0.05 and a 95% confidence interval (CI) of the differences was calculated to estimate the effect size for all statistical comparisons.

## RESULTS

### Maximal Torque, Motor Unit Decomposition, and Tracking during Isometric Contractions

**Figure 3.**
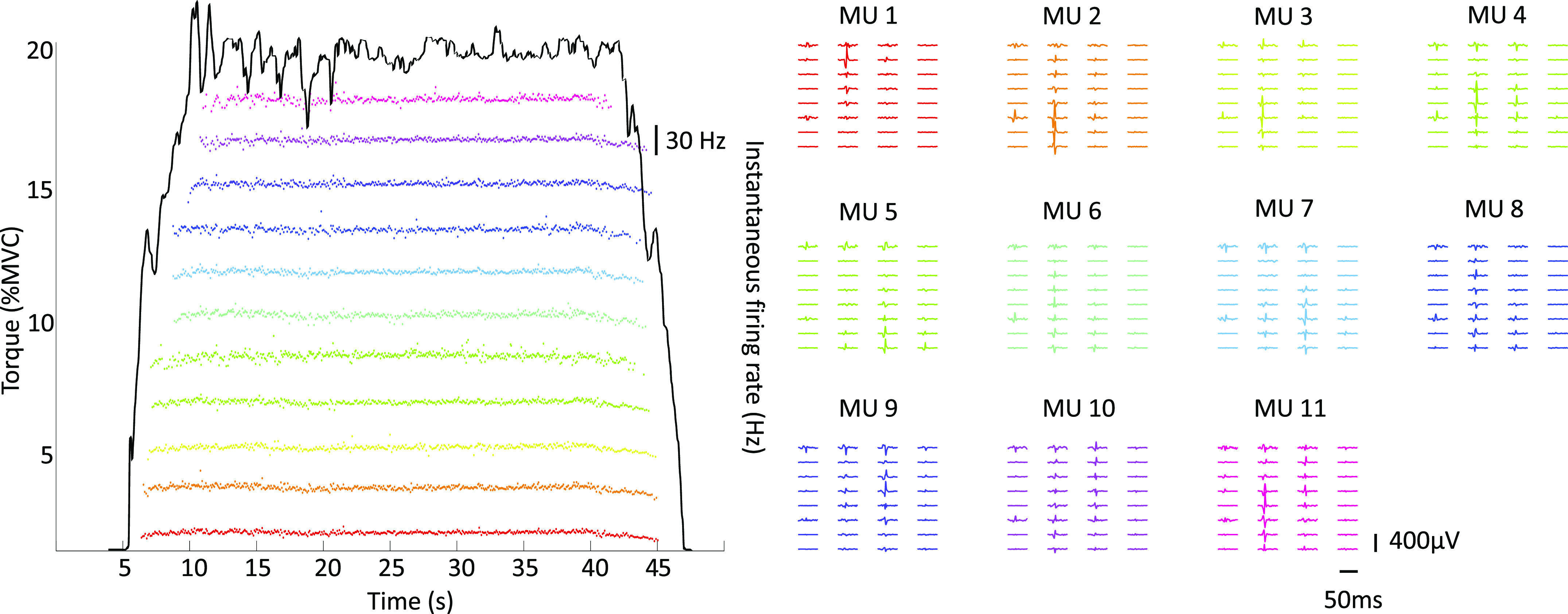
Motor unit (MU) identification during isometric contractions. A total of 11 motor units (MUs) were decomposed from the high-density surface electromyography (HDEMG) signals in a representative participant (*n* = 1, male) during an isometric contraction at 20% of the maximum voluntary torque (0° of plantarflexion). Instantaneous firing rate with torque profile can be seen on the left of the figure while two-dimensional (2D) motor unit action potentials (MUAPs) from each of these motor units can be seen on the right of the figure. Each waveform corresponds to one EMG channel, and the action potentials belonging to each of these units can be seen spread across the 32 EMG channels of the grid. Note the clear differences in MUAP shape for each of the identified units.

**Figure 4.**
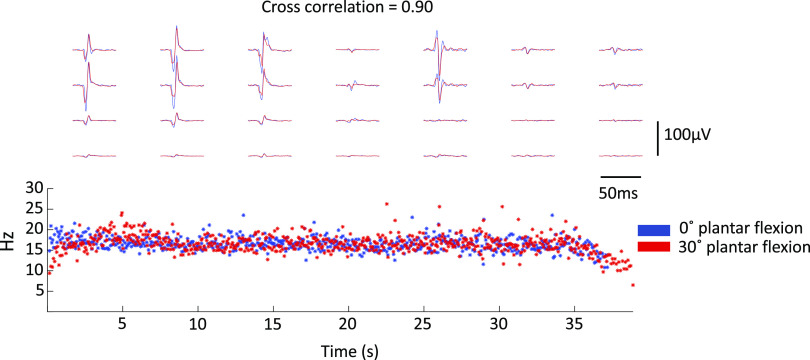
Motor unit tracking. A representative example of a motor unit that was tracked across two-plantarflexion angles at 20% maximum voluntary contraction (MVC) can be seen in the figure (*n* = 1, male participant). Each waveform corresponds to one single-differential electromyography (EMG) channel, and the action potentials belonging to each of these units can be seen spread across the 28 EMG channels of the grid. The motor unit action potentials obtained from the recordings made across the two joint angles (blue, 0° of plantar flexion and red, 30° of plantar flexion) were then matched by cross-correlation as seen previously (42). For this motor unit, the action potentials had a cross correlation coefficient of 0.90 across angles. The instantaneous firing rate of this unit can be seen on the bottom of the figure.

The MVC dorsiflexion torque differed at the two ankle positions and was 25.2 (13.2) N·m at the short (0° plantar flexion) TA length and 51.8 (12.5) N·m at long (30° plantar flexion) TA length (*P* < 0.001). During both sustained and sinusoidal isometric contractions, the average number of motor units identified per participant was 7 (3) at 20% MVC-0°, 7 (2) at 20% MVC-30°, 7 (3) at 40% MVC-0°, and 6 (2) at 40% MVC-30°. A representative example of the decomposition results obtained during a 20% MVC contraction at the short TA length (0° of plantar flexion) from one participant can be seen in Fig. 3. In this example, each motor unit has a distinct MUAP shape (right side of the figure) that allowed accurate identification of discharge times (left side of the figure). An average of 3 (2) motor units could be tracked successfully per participant for the two muscle lengths [2D MUAP cross-correlation coefficient 0.83 (0.03)] and 3 (1) motor units per participant for the two torque levels [2D MUAP cross-correlation coefficient 0.88 (0.05)]. A representative example of the tracking procedure for the two joint angles with the HDEMG-US grids can be seen in Fig. 4.

### Relationships between Fascicle Length, Torque, and Motor Unit Discharge Rate during Isometric Contractions

Absolute fascicle lengths and pennation angles at rest and during isometric contractions at the two different target torques and two joint angles are presented in Table 1. Overall, when TA was lengthened, pennation angles decreased and fascicle length increased. In addition, TA pennation angles increased with increasing dorsiflexion torque (torque effect: *P* = 0.018, mean difference = −1.5°, 95% CI = −2.7° to −0.3°) and TA fascicle length decreased with increasing dorsiflexion torque (torque effect: *P* < 0.001, mean difference = −3.2 mm, 95% CI = −4.3 mm to −2.0 mm) at both muscle lengths.

**Table 1.** Tibialis anterior muscle, fascicle length, and pennation angles at rest and during sustained isometric dorsiflexion contractions at 20% and 40% MVC at short and long muscle lengths (different joint angles)

	Short (0° Plantar Flexion)	Long (30° Plantar Flexion)	Mean Difference [95% Confidence Interval]	*P* Value
Pennation angle, °, rest	15.2 (1.7)	12.0 (1.7)	3.2 [1.0 to 5.4]	<0.001
Fascicle length, mm, rest	60.2 (8.8)	74.5 (9.1)	−14.3 [−18.9 to −9.5]	<0.001
Pennation angle, °, 20% MVC	19.2 (3.7)	16.9 (3.9)	2.2 [0.9 to 3.5]	0.001
Fascicle length, mm, 20% MVC	52.7 (7.5)	66.3 (9.4)	−13.5 [−19.6 to −7.5]	<0.001
Pennation angle, °, 40% MVC	20.8 (3.1)	17.4 (4.0)	2.6 [1.0 to 4.2]	0.001
Fascicle length, mm, 40% MVC	50.8 (6.9)	61.9 (7.9)	−11.1 [−15.3 to −7.0]	<0.001

Values are reported as means (SD). Mean difference between lengths is reported with the 95% confidence interval (CI) of the difference. MVC, maximum voluntary contraction; TA, tibialis anterior.

**Figure 5.**
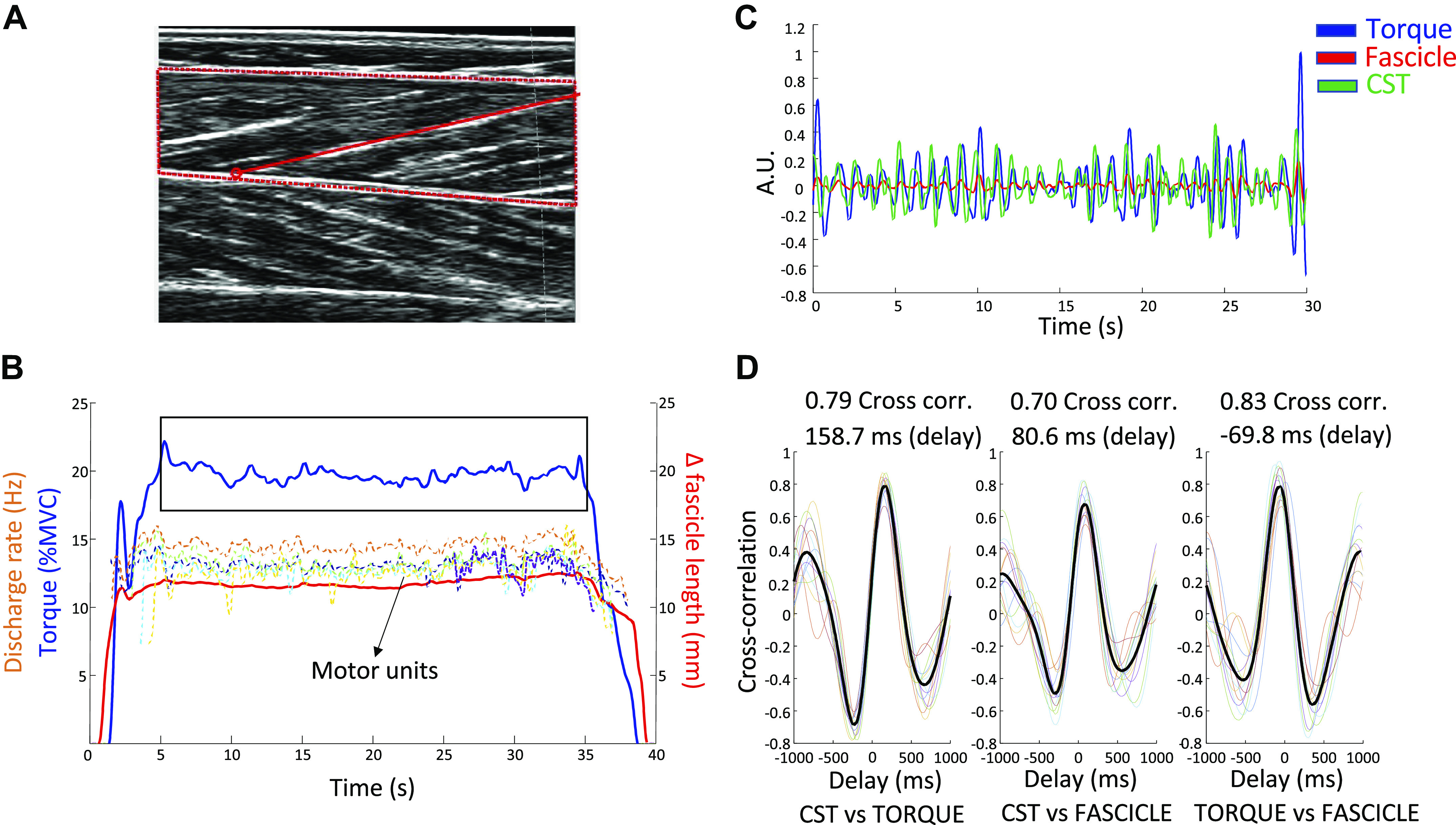
Fascicle length tracking procedure and correlation with torque and motor unit data (*n* = 1, male participant). *A*: tibialis anterior muscle ultrasound image and a fascicle of interest is highlighted in red. *B*: the length data obtained from the tracking of this fascicle was then correlated with torque and cumulative spike train (CST) signals. Fascicle length data is presented as the amount of shortening from rest to target torque (fascicle length during rest minus fascicle length reached at target torque). Note that fascicle shortening precedes the generation of torque during the ramp-up phase of the contraction and then returns to baseline values after the torque signal returns to zero. The steady torque part that was selected to correlate signals is inside the rectangle. Colored dashed lines represent the smoothed firing rate of the five motor units used to calculate the CST. *C*: common fluctuations from the three generated signals (torque, CST, and fascicle length) in the steady-torque part of the contraction (segment inside the rectangle in *B*). *D*: cross-correlation and lag (delay, ms) results between CST vs. torque, CST vs. fascicle length (fascicle), and fascicle vs. torque. Colored lines represent the cross-correlation of twelve 5-s overlapped segments (50% overlap) and the black line is the average cross-correlation of all segments.

**Figure 6.**
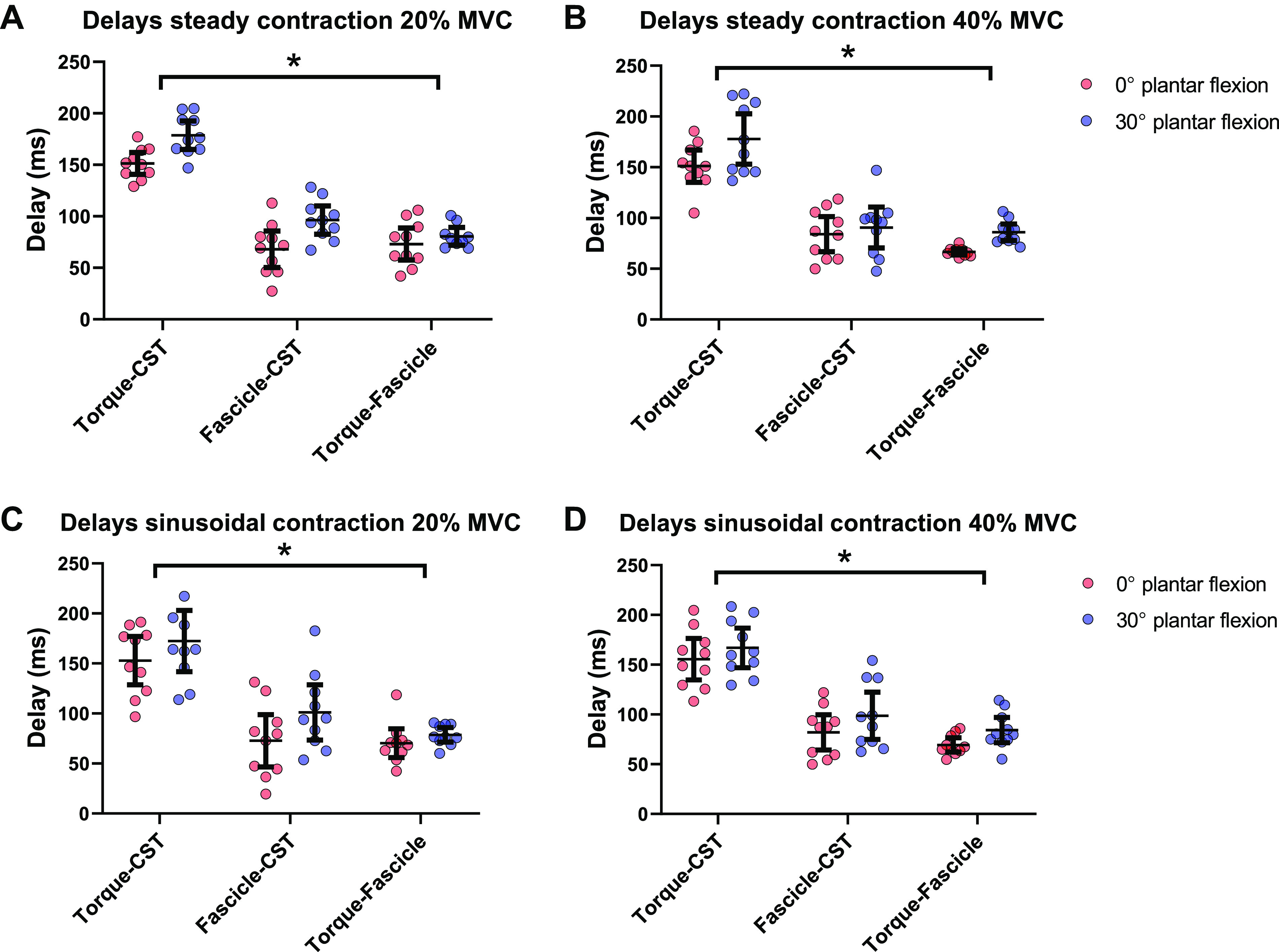
Cross-correlation delays during sustained and sinusoidal isometric contractions. Delays between cumulative spike train (CST) vs. torque, CST vs. fascicle length (fascicle) and fascicle vs. torque can be seen for sustained contractions at 20% maximum voluntary contraction (MVC) (*A*) and 40% MVC (*B*), and sinusoidal contractions at 20% MVC (*C*) and 40% MVC (*D*) during 0° and 30° of plantarflexion. Dots represent individual values (*n* = 10, male participants), whiskers represent the mean and 95% confidence interval. A three-way repeated-measures ANOVA was used for statistical comparisons.*Significant effect of joint angle (*P* < 0.05). A summary of statistical comparisons for delays can be seen in Table 4.

The tracking of individual motor units across short- and long muscle lengths revealed similar discharge rates at the different joint angles, 20% MVC 0° plantar flexion: 15.3 (2.6) Hz, 20% MVC 30° plantar flexion: 14.7 (1.4) Hz, 40% MVC 0° plantar flexion: 16.7 (2.3) Hz, 40% MVC 30° plantar flexion: 17.0 (2.4) Hz (muscle length effect: *P* = 0.74, mean difference = 0.1 Hz, 95% CI = −1.2 to 1.5 Hz). However, discharge rate increased with increasing torque (torque effect: *P* = 0.006, mean difference = −1.8 Hz, 95% CI = −3.0 to −0.7 Hz). A representative example of the concurrent assessment of changes in fascicle length, torque, and CST can be seen in Fig. 5. Cross correlation results are presented in Table 2. The statistical comparisons of the cross-correlation results obtained when assessing torque versus CST, fascicle length versus CST, and torque versus fascicle length across the two joint positions and torques can be seen in Table 3. Overall, mean cross-correlation coefficient values ranged from moderate to high (>0.50) showing that there was a relationship between CST, fascicle length, and torque. It is worth noting that all correlation coefficients were higher during the sinusoidal isometric contractions (contraction type effect: *P* = 0.001, mean difference = −0.11, 95% CI = −0.16 to −0.06). Cross-correlation delays (Fig. 6) during sustained isometric contractions at 30° of plantar flexion (long muscle lengths) were larger than those at 0° plantar flexion (short muscle lengths) at both torque levels in all the comparisons of the generated signals, in both sustained and sinusoidal isometric contractions, with no differences between sustained versus sinusoidal isometric lag values (see Table 4 for a summary of main delay effects).

**Table 2.** Cross-correlation coefficients comparing common modulations in cumulative spike train vs. torque, CST vs. fascicle length, and torque vs. fascicle length during isometric contractions

	Short TA Length 20% MVC		Long TA Length 20% MVC		Short TA Length 40% MVC		Long TA Length 40% MVC	
	Means (SD)	Range	Means (SD)	Range	Means (SD)	Range	Means (SD)	Range
Steady isometric contractions								
Torque vs. CST	0.74 (0.07)	0.65–0.88	0.74 (0.07)	0.62–0.85	0.74 (0.08)	0.63–0.85	0.61 (0.15)	0.31–0.78
Fascicle vs. CST	0.64 (0.09)	0.51–0.81	0.57 (0.18)	0.31–0.75	0.63 (0.15)	0.33–0.85	0.50 (0.13)	0.31–0.66
Torque vs. fascicle	0.71 (0.14)	0.40–0.88	0.67 (0.19)	0.33–0.89	0.78 (0.2)	0.33–0.93	0.72 (0.15)	0.50–0.91
Oscillatory isometric contractions								
Torque vs. CST	0.78 (0.1)	0.66–0.89	0.77 (0.08)	0.62–0.83	0.77 (0.07)	0.66–0.89	0.66 (0.11)	0.45–0.82
Fascicle vs. CST	0.71 (0.1)	0.58–0.86	0.73 (0.06)	0.69–0.78	0.74 (0.08)	0.60–0.86	0.62 (0.13)	0.41–0.75
Torque vs. fascicle	0.87 (0.07)	0.75–0.95	0.89 (0.07)	0.75–0.95	0.91 (0.06)	0.80–0.96	0.89 (0.06)	0.77–0.96

Values are reported as means (SD) and min-max range. CST, cumulative spike train; MVC, maximum voluntary contraction; TA, tibialis anterior.

**Table 3.** Pairwise comparisons between cross-correlation coefficient results during steady and sinusoidal contractions

Cross-Correlation Comparison		Mean Difference [95% Confidence Interval]	*P* Value
Steady contractions			
Torque vs. CST	CST vs. fascicle	0.13 [0.02 to 0.24]	0.02
Torque vs. CST	Torque vs. fascicle	−0.01 [−0.12 to 0.10]	0.85
CST vs. fascicle	Torque vs. fascicle	−0.14 [−0.25 to −0.03]	0.01
Sinusoidal contractions			
Torque vs. CST	CST vs. fascicle	0.05 [−0.01 to 0.10]	0.069
Torque vs. CST	Torque vs. fascicle	−0.14 [−0.19 to −0.09]	<0.001
CST vs. fascicle	Torque vs. fascicle	−0.18 [−0.23 to −0.13]	<0.001
Steady vs. sinusoidal contractions			
Torque vs. CST steady	Torque vs. CST sinusoidal	−0.04 [−0.10 to −0.01]	0.049
CST vs. fascicle steady	CST vs. fascicle sinusoidal	−0.12 [−0.21 to −0.09]	0.002
Torque vs. fascicle steady	Torque vs. fascicle sinusoidal	−0.16 [−0.23 to −0.09]	<0.001

Mean differences are calculated in order of presentation (column 1 minus column 2). CST, cumulative spike train

**Table 4.** Pairwise comparisons for between-signal delays

Main Effects		Mean Difference [95% Confidence Interval], ms	*P* Value
Steady contractions			
0°	30°	−18.59 [−28.62 to −8.56]	0.002
Torque vs. CST	CST vs. fascicle	79.99 [68.78 to 91.20]	<0.001
Torque vs. CST	Torque vs. fascicle	89.26 [78.05 to 100.46]	<0.001
CST vs. fascicle	Torque vs. fascicle	9.27 [−1.94 to 20.47]	0.127
Sinusoidal contractions			
0°	30°	−17.31 [−30.27 to −4.35]	0.014
Torque vs. CST	CST vs. fascicle	74.40 [59.50 to 89.30]	<0.001
Torque vs. CST	Torque vs. fascicle	87.38 [72.48 to 102.29]	<0.001
CST vs. fascicle	Torque vs. fascicle	12.98 [−1.92 to 27.89]	0.10

Mean differences are calculated in order of presentation (column 1 minus column 2). CST, cumulative spike train.

### Variations in TA Fascicle Length and Mean Recruitment/de-Recruitment Thresholds during Isometric Contractions

**Figure 7.**
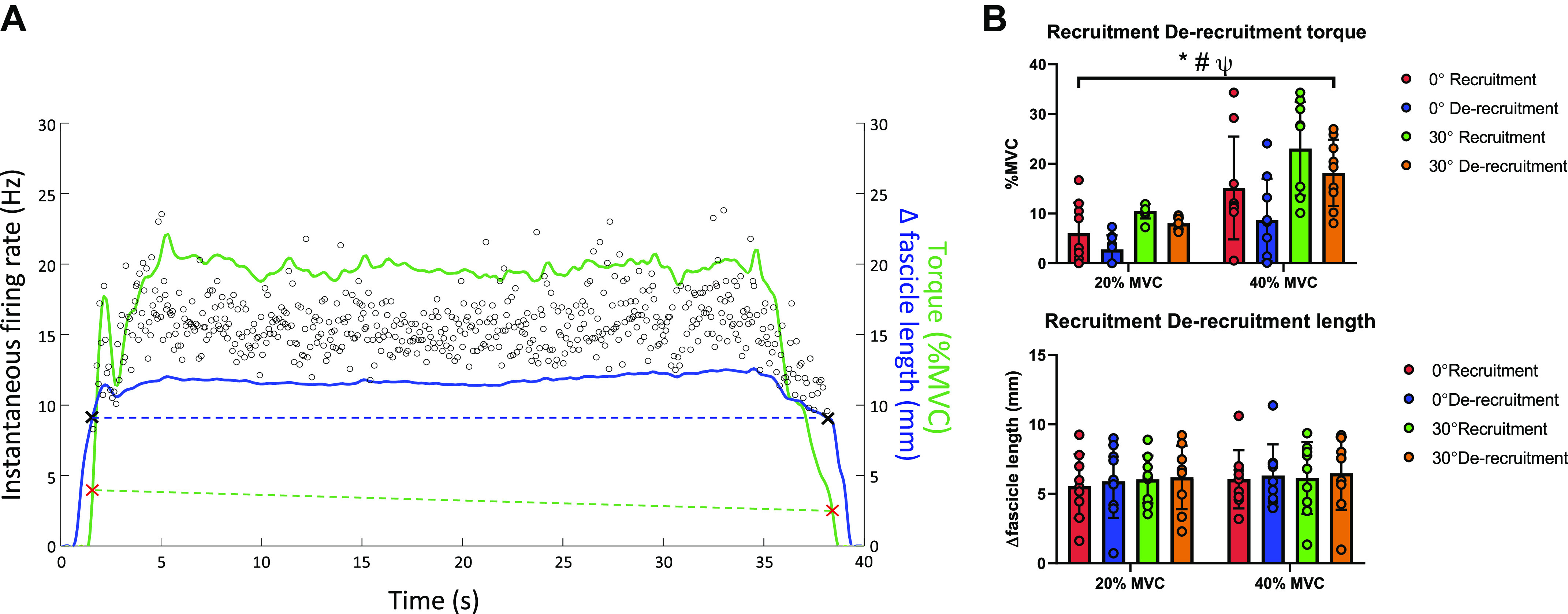
Recruitment and de-recruitment thresholds in relation to torque and fascicle length. Representative example of recruitment and de-recruitment threshold of a motor unit in relation to torque and fascicle length can be seen *A*. The figure shows that the differences between recruitment and de-recruitment thresholds were greater when quantified as % maximum voluntary contraction (MVC) torque (green dashed line, red crosses represent recruitment and de-recruitment thresholds in %MVC) compared with those obtained from Δfascicle length (blue dashed line, black crosses represent recruitment and de-recruitment thresholds in fascicle length). Fascicle length data are presented as the amount of shortening from rest to target torque (fascicle length during rest-fascicle length reached at target torque). The same results are evident at the group level (*B*) as recruitment thresholds are consistently higher than de-recruitment thresholds across target torques and angles when considered as %MVC torque (*top right*) but similar when calculated from fascicle length data (*bottom right*). Bar heights represent the mean values for the group, dots represent individual values (*n* = 10, male participants), whiskers represent the 95% confidence interval. A three-way repeated-measures ANOVA was used for statistical comparisons.*Significant effect of recruitment-de-recruitment (*P* < 0.05). #Significant effect of joint angle (*P* < 0.05). ΨSignificant effect of torque (*P* < 0.05). A summary of statistical comparisons for recruitment, de-recruitment thresholds can be seen in Table 5.

Mean motor unit recruitment and de-recruitment thresholds in terms of both Δ fascicle length (mm) and torque (%MVC) are shown for a representative participant in Fig. 7*A*. Mean motor unit de-recruitment thresholds were consistently lower than the mean recruitment thresholds when assessed in terms of %MVC torque but similar when quantified as Δ fascicle length (Fig. 7*B*). In addition, although mean recruitment and de-recruitment thresholds increased in terms of %MVC torque across 20% and 40% MVC levels, on average, motor units were recruited and de-recruited at a similar Δ fascicle length with increasing torque. Finally, mean recruitment-de-recruitment thresholds were higher at long muscle lengths (0° plantar flexion vs. 30° plantar flexion) when considered as %MVC torque but not in terms of Δ fascicle length. Main effects for mean recruitment, de-recruitment threshold comparisons when expressed in %MVC and Δfascicle length can be seen in Table 5.

**Table 5.** Pairwise comparisons for mean recruitment thresholds expressed in %MVC torque or fascicle length

Main Effects		Mean Difference [95% Confidence Interval] %MVC	*P* Value
Recruitment, de-recruitment in %MVC			
Recruitment	De-recruitment	3.93 [0.50 to 7.36]	0.03
20% MVC	40% MVC	−8.10 [−11.42 to −4.77]	<0.001
0°	30°	−10.70 [−15.98 to −5.42]	0.001
Recruitment, de-recruitment in fascicle length, mm			
Recruitment	De-recruitment	−0.08 [−0.75 to 0.60]	0.81
20% MVC	40% MVC	−0.40 [−1.41 to 0.60]	0.39
0°	30°	−0.29 [−2.17 to 1.58]	0.73

Mean differences are calculated in order of presentation (column 1 minus column 2). MVC, maximum voluntary contraction.

## DISCUSSION

This study shows that modulations in TA motor unit discharge rate are closely related to both changes in TA fascicle length and dorsiflexion torque during isometric contractions. These relationships allowed us to quantify the delays between motor unit firing activity and fascicle shortening as well as fascicle shortening and joint torque during voluntary contractions. We demonstrated that delays between the neural drive and fascicle length, or the generated torque, are larger when the muscle contracts isometrically at longer fascicle lengths. In addition, due to the feasibility of tracking motor units across different muscle lengths and target torques, we were also able to show that mean recruitment and de-recruitment thresholds for TA are similar when considered in terms of its fascicle shortening length, meaning that motor units are recruited and de-recruited at the same change in fascicle length, regardless of the joint position or torque exerted. Taken together, the examination of these relationships allows for an accurate assessment of the conversion of neural activity into muscle contractions.

### CST, Fascicle Length, and Torque Relationships

A number of studies have shown that fluctuations in firing rate are closely related to the fluctuations in torque or force (16, 38, 40). Considering this observation, we attempted to quantify the level of correlation between fluctuations in firing rate (CST), changes in fascicle length and torque, to first understand whether the firing properties obtained from the identified motor units was linked to the fascicles from the region of interest (CST vs. fascicle length correlation) and also to confirm that motor unit firings identified with HDEMG-US grids would be able to predict the fluctuations in torque transmitted via the tendon. The findings showed moderate to high levels of correlation of all signals, confirming that the motor unit data provided a good representation of the changes in TA fascicle length from the region of interest. Moreover, all correlation levels were stronger when greater torque variability was induced via sinusoidal isometric contractions. That result showed that motor unit activity is modulated together with changes in fascicle length, which further confirmed the close relationship between motor unit discharge and fascicle length changes.

It is important to mention that this greater variability did not affect cross-correlation delays as similar delays were observed in sustained and oscillatory contractions. Recent studies correlating both motor unit firings and torque have shown that the delay between the CST and torque decreases at higher contraction velocities (40, 41). It is likely that the frequency at which the participants matched the sinusoidal target (0.5 Hz) was similar to the involuntary torque modulations induced during sustained isometric contractions. Indeed, both Del Vecchio et al. (40) and Martinez-Valdes et al. (41) found even larger delays between CST and torque (∼300 ms) when the contractions were modulated at slow frequencies (0.5 and 0.25 Hz). The delay between CST and torque obtained in our study (∼150 ms) was shorter than those reported previously, possibly due to the differences between dynamometers (i.e., Biodex System 3 vs. Humac Norm) (43). This approach used here provides an advantage in assessing changes in neural activity in relation to muscle contractile dynamics, as these measurements are not affected by potential dynamometer differences and could also be used to isolate individual muscle dynamics for joints where multiple muscles actuate the joint. However, the effect of different dynamometers on the measurement of delays needs to be investigated further.

### CST, Fascicle Length, and Torque Delays

Historically, studies aiming to quantify the time required to convert neural activity into mechanical output calculated the time difference between the onset of force/torque and muscle activation (42) during both voluntary and electrically induced contractions (electromechanical delay). Measurement of the electromechanical delay commonly neglects the lag between the generation of a contraction and the transmission of force to the tendon, and then transmission to the measuring apparatus [which generates great variability in results (44)]. More recent studies have aimed to quantify these delays by combining conventional bipolar EMG or HDEMG amplitude and ultrasound (17, 45). Similar to the current study, previous research also revealed that fascicle shortening happens before force/torque is generated (17, 18, 45). However, the magnitude of these delays is considerably smaller than the ones presented herein, with ∼6 ms (EMG-fascicle length) and ∼12 ms (EMG-force) of delay for electrically induced contractions (18, 46) and ∼29 ms (EMG-fascicle length) and ∼50 ms (EMG-force) of delay for voluntary contractions (17).

Besides the multiple methodological limitations of the aforementioned approaches, such as issues with the correct detection of the onset of muscle activation from EMG amplitude (47, 48) and weak correlations between EMG amplitude and force (16), it is difficult to transfer the information obtained from these delays to functional contractions. Moreover, estimates of the electromechanical delay are calculated at a single time instant when the muscle activates from a passive state, neglecting the influence of the muscle’s afferent sensors (i.e., muscle spindle, Golgi tendon organ), active motor unit twitch properties, and active/passive muscle tissue interactions responsible for the regulation of muscle force during contractions (49). In contrast, the cross-correlation of the signals generated from HDEMG-US motor unit decomposition and ultrasound provides a better estimation of these delays, as such assessment considers mechanisms responsible for contraction dynamics (i.e., fascicle length responses to firing rate modulations) rather than just assessing delay differences between the measured signals at contraction onset.

The longer delays reported in our study are likely due to twitch characteristics of the active motor units (40), changes in MUAP duration, and afferent input received during the contraction (50, 51). Regarding motor unit twitches, Cudicio et al. (49) used a new technique of motor unit twitch estimation (52) and reported values of motor unit twitch time-to-peak (∼110 ms) similar to the delays between CST and torque reported in our study. In terms of afferent input, it has been previously shown that both Golgi tendon organs and muscle spindles discharge continuously during isometric contractions (50, 51). This sensory information helps to regulate muscle force but could potentially induce delays in the conversion of neural activity into muscle contraction.

### CST, Fascicle Length, and Torque Delays at Different Joint Positions

Several studies have reported that muscle twitches have a longer duration (greater contraction and half-relaxation times) when the muscle is lengthened (49, 53, 54). Therefore, it is very likely that an increase in the overall duration of the motor unit responses at longer lengths is the reason for the more delayed muscle contraction and transmission of force to the tendon observed at the two submaximal torque targets (20% and 40% MVC). In addition, changes in tendon and aponeurosis elasticity during lengthening could also reduce the amount of neural drive required to activate the muscle (55–57) and potentially increase the delays measured in the current study. Nevertheless, the assessment of both motor unit contractile properties and the effect of passive structural properties of the muscle on motor unit behavior needs to be confirmed in future investigations.

### Mean Recruitment versus De-recruitment Threshold Discrepancies

Motor unit studies typically use torque/force to assume changes in muscle behavior, however, delays between motor unit firing and torque due to force transmission delays (between muscle, tendon, and dynamometer) could create offsets when comparing recruitment (torque rise) versus de-recruitment (torque drop) thresholds. The findings from this study suggest that it is important to consider these delays when interpreting recruitment and de-recruitment results from torque or force signals since we were able to observe that mean recruitment and de-recruitment thresholds differed when quantified as %MVC torque but were similar when quantified in terms of fascicle length. Moreover, we were able to observe that the length at which a motor unit was recruited and de-recruited was maintained across joint angles and torque levels. Fascicle shortening preceded torque generation, but fascicles returned to resting values after the dorsiflexion torque returned to baseline (0 N·m). This means that during the ramp-down phase of the contraction, there is a time-point where the muscle returns passively to its original length. This can explain why many studies show lower torque values for de-recruitment than recruitment when considered as %MVC torque or absolute torque (58–60) and emphasizes the need to interpret recruitment versus de-recruitment relationships with caution, particularly when quantified from the torque measured at the dynamometer. Interestingly, similar divergences were recently reported when considering recruitment and de-recruitment in terms of joint angle or fascicle length during isometric plantar flexion contractions at varying knee joint angles (20).

### Limitations and Future Developments

In this study, we were able to identify an average of seven motor units across contractions, which is lower than the number of units identified with nontransparent HDEMG electrodes on TA (59). These differences can be attributed to a number of factors. First, the grid of electrodes used in the current study contained 32 electrodes versus the 64 electrodes conventionally used to decompose EMG signals. It has been shown that a larger number of channels enhances the spatial identification of MUAPs and therefore improves the separation of the multiple motor unit sources from the HDEMG with blind-source separation methods (26, 61). Second, the interelectrode distance of the HDEMG-US grid (10 mm) is less selective than the 64-channel grids (8 mm) commonly used to decompose motor unit activity, which can again influence the separation of MUAPs from the HDEMG. Therefore, improvements in electrode construction will likely increase the number of motor units identified with HDEMG-US.

The depth at which we tracked the fascicles (superficial portion of the TA) versus region of motor units recorded may also contribute to discrepancies in correlations between the generated signals. HDEMG-US electrodes most likely only detect motor unit activity of the most superficial motor units (62). Therefore, the contribution of deeper motor units may have been missing from our calculations. Nevertheless, it is important to mention that, on average, the cross-correlation between motor unit discharge rate and torque was high (∼0.70) and similar to the values reported in previous studies (16, 38, 40).

Muscle fascicle imaging using ultrasound also has some limitations: the resolution of the image could limit the tracking of small changes in fascicle length, only one plane of the muscle can be imaged at a time and only a single fascicle was tracked. Improvements in ultrasound probe construction and/or the addition of another ultrasound probe in a different portion of the electrode grid would likely help to improve estimations of changes in fascicle length during isometric contractions. The accurate assessment of motor unit and fascicle data across torque levels and joint positions opens up the opportunity to explore more diverse isometric contraction models (i.e., ballistic contractions or faster sinusoidal contractions) and shortening and lengthening contractions. Our present results were not affected by more dynamic variations in isometric torque and the tracking of motor units across joint angles revealed that MUAPs did not change substantially within the range of motion investigated. It is important to note that all cross-correlation coefficients were higher during sinusoidal contractions. Greater variations in muscle force during sinusoidal contractions could have improved the detection of fascicle movement with ultrasound imaging, which could facilitate the identification of relationships between signals. Therefore, this contraction type could be preferred when it is difficult to visualize the shortening of fascicles during a steady contraction (i.e., individuals with high force steadiness).

Very recent studies using HDEMG recordings (without ultrasound) have successfully identified motor units during shortening and lengthening contractions (63, 64), therefore, it is very likely that future studies assessing changes in motor unit activity and fascicle length will be able to assess these interactions during dynamic conditions. Finally, it is important to mention that only male participants were included in this study due to the low motor unit yield commonly observed in females with HDEMG recordings (65). Future studies should aim to test the accuracy of HDEMG-US recordings in females to provide a wider representation of these results across genders.

### Conclusions

This study shows that modulations in motor unit firing rate are closely related to changes in fascicle length during isometric contractions. The determination of these relationships allows quantifying delays between MUAP propagation, muscle contraction, and subsequent transmission of force to the tendon during sustained and functional torque modulation, providing a better representation of the mechanisms responsible for the control of muscle force during active contractions.

## DATA AVAILABILITY

The data and analysis code are available from the corresponding author, E.M.-V, upon reasonable request.

## GRANTS

This study was partially funded with the University of Birmingham International Engagement Fund (BIEF) (to E. Martinez-Valdes).

## DISCLOSURES

No conflicts of interest, financial or otherwise, are declared by the authors.

Glen Lichtwark is an editor of *Journal of Applied Physiology* and was not involved and did not have access to information regarding the peer-review process or final disposition of this article. An alternate editor oversaw the peer-review and decision-making process for this article.

## AUTHOR CONTRIBUTIONS

E.M.-V. and F.N. conceived and designed research; E.M.-V. and P.A.P. performed experiments; E.M.-V. and F.N. analyzed data; E.M.-V., F.N., A.B., P.A.P., D.F., G.A.L., and A.G.C. interpreted results of experiments; E.M.-V. prepared figures; E.M.-V. drafted manuscript; E.M.-V., F.N., A.B., P.A.P., G.L.C., D.F., G.A.L., and A.G.C. edited and revised manuscript; E.M.-V., F.N., A.B., P.A.P., G.L.C., D.F., G.A.L., and A.G.C. approved final version of manuscript.
